# Oral and Periodontal Manifestations in Systemic Sclerosis: A Comprehensive Review of Pathophysiology, Clinical Features, and Management Strategies

**DOI:** 10.3290/j.ohpd.c_2825

**Published:** 2026-09-10

**Authors:** Rayan Meer

**Affiliations:** a Rayan Meer Assistant Professor, Department of Preventive Dental Sciences, College of Dentistry, Taibah University, Medinah, Saudi Arabia. Design and conception of the study, data analysis, interpretation, and manuscript preparation.

**Keywords:** autoimmune diseases, periodontal disease, scleroderma oral manifestations, systemic sclerosis, xerostomia.

## Abstract

**Purpose:**

Systemic sclerosis is a connective tissue disorder that presents with various orofacial manifestations, which may influence oral health status and complicate dental management. This review sought to identify the oral and periodontal signs linked to systemic sclerosis, examine the specific oral health challenges faced by individuals with this condition, and outline their potential oral healthcare requirements.

**Materials and Methods:**

A review of the available literature was undertaken to evaluate the reported oral and periodontal manifestations associated with systemic sclerosis and to assess their clinical implications.

**Results:**

Orofacial manifestations of systemic sclerosis include skin fibrosis, reduced mouth opening, dry mouth, and difficulty swallowing, as well as a potential increased risk of tooth decay, periodontal disease, and oral cancer. These oral and periodontal characteristics, along with their associated complications, improve the understanding and anticipation of oral healthcare requirements for affected patients.

**Conclusion:**

Recognition of the oral and periodontal features of systemic sclerosis emphasizes the importance of raising awareness among dental professionals to enhance treatment planning and improve the quality of dental care delivered to this patient population.

Scleroderma and systemic sclerosis (SSc) are autoimmune disorders affecting multiple systems, which adversely influence both the oral health and the ability of individuals with SSc to obtain dental care. While only a few reports of the disease and its oral features have been described, this review aims to examine the orofacial complications and provide an updated literature review of the periodontal and oral manifestations associated with systemic sclerosis.

SSc has an incidence of 10–20 cases per million annually, which may increase due to improved awareness and diagnostic methods, as well as environmental or occupational factors that induce scleroderma-like conditions.^18,34^ Early diagnosis of systemic sclerosis disease may probably increase the disease prevalence and also the patients’ survival rates. A prevalence of 1 to 15/100,000 has been reported for SSc in different geographical regions.^36^ Comparable prevalence rates have been reported in various countries and regions such as the United States, Europe, Argentina, and Australia, ranging between 150 to 300 cases per million, while lower estimates are observed elsewhere, e.g., the United Kingdom, Japan, India, Taiwan, and Scandinavia.^5^ Death rates vary according to both sex and the specific organ affected, with males experiencing higher mortality.^30^ The survival rate stands near 85% at five years but declines to under 70% by the ten-year mark.^36^ The American College of Rheumatology (ACR) and the European League Against Rheumatism (EULAR) developed the criteria of the new classification system that deals with three main factors: presence of fibrosis in skin and/or internal organs, production of specific autoantibodies, and vasculopathy presence. Under the updated classification criteria, a diagnosis of SSc is established when a patient exhibits finger-skin thickening that spreads proximally beyond the metacarpophalangeal joints. In the absence of the major criterion, seven other characteristics are scored, such as digital skin thickening, Raynaud’s phenomenon, telangiectasia, fingertip lesions, nailfold capillary abnormalities, pulmonary hypertension or interstitial lung disease, and scleroderma-specific autoantibodies—each of which is assigned a point score, where a score of ≥9 is definitive of SSc.^38^ Diagnosis of systemic scleroderma is mostly on the basis of recognizing characteristic features such as Raynaud’s phenomenon, digital puffiness, nailfold capillary abnormalities on capillaroscopy, and detection of disease-specific serum autoantibodies.

As mentioned before, SSc is a multi-organ disease with different clinical presentation, but commonly affected the skin in early phases in addition to internal-organ involvement. Careful clinical examination is always needed with other investigative means to distinguish systemic sclerosis from other autoimmune diseases of connective tissue, as it may overlap with other syndromes.

## Literature Search Strategy

A narrative review was conducted to identify publications addressing oral and periodontal manifestations of systemic sclerosis. Electronic searches were performed in June 2025 using PubMed/MEDLINE, Scopus, Web of Science and Google Scholar. The following terms were used in the search: “systemic sclerosis”, “scleroderma”, “oral manifestations”, “orofacial manifestations”, “periodontal disease”, “xerostomia” and “periodontal ligament”. Articles written in English from January 1980 until June 2025 were considered for inclusion. Reference lists of selected articles were manually checked for additional relevant studies. The oral, periodontal, radiological, and clinical management of systemic sclerosis was included in the publications reviewed.

## Clinical Features of Oral Manifestations and Management Strategies

Systemic health is closely linked to oral health, with periodontal disease frequently associated with systemic pathological changes. Importantly, studies have shown that periodontal disease may be an etiological factor in the development of diabetes mellitus, preterm low birth weights, and cardiovascular atherosclerotic disorders.^19,20^

**Fig 1:**
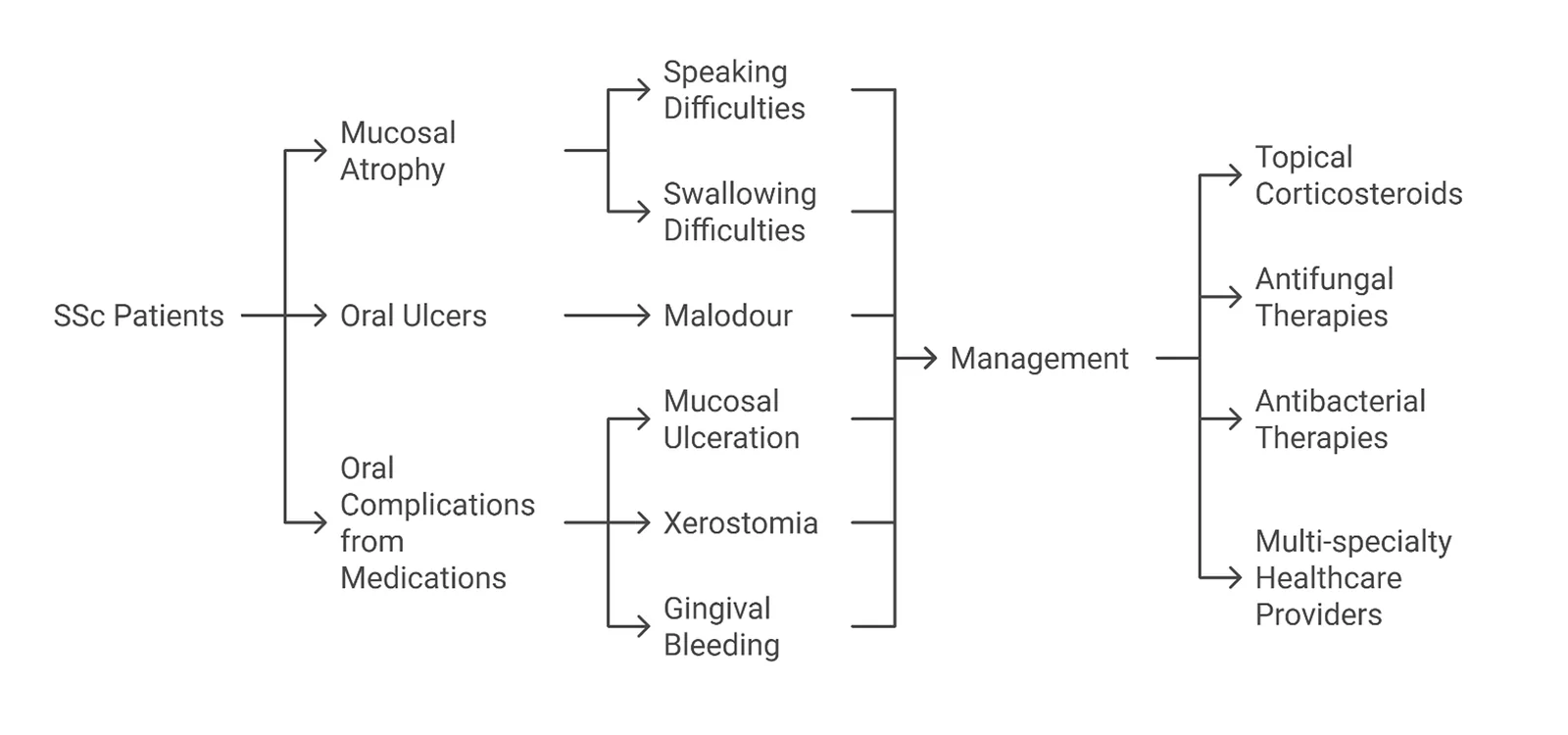
Causes of oral signs and symptoms of SSc patients and its management.

Research indicates that between 80% and 90% of individuals diagnosed with systemic sclerosis (SSc) experience orofacial manifestations. These commonly include skin fibrosis, reduced mouth opening (microstomia), dry mouth (xerostomia), and difficulty swallowing (dysphagia) (Fig 1). Additionally, these patients may face an increased susceptibility to dental caries, periodontal diseases, and oral malignancies.^22,35,39^ Most affected people have multiple disease-related characteristics, which can negatively affect a patient’s emotional and social life as well as their functional and esthetic status.^1,24^ The progression of the disease is often variable and unpredictable, with the orofacial manifestations differing depending on the specific subtype of systemic sclerosis.^25,33^ Moreover, systemic symptoms, including musculoskeletal discomfort, persistent fatigue, diminished fine motor skills, swallowing difficulties, and involvement of the heart and lungs can impair patients’ capacity to manage their oral hygiene effectively and postpone seeking professional dental treatment. This could potentially limit their ability to receive routine or necessary oral health care.^2,40^

## Reduced Mouth Opening

The majority of SSc patients have oral symptoms (52.0 – 80%), and patients’ quality of life is significantly impacted by reduced mouth opening since it affects several activities, including eating, speaking, maintaining good oral hygiene, and good esthetics. This is mostly due to the disease’s clinical features, which include increased collagen deposition in the orofacial tissues.^4,27^ Tightening of the facial muscles and skin restricts the ability to open the mouth fully. Reduced mouth opening has been associated with mandibular bone erosions in patients with SSc. This restriction has been associated with elevated incidences of tooth loss, more severe periodontal disease, and an overall worsening of oral status.^6,7^

**Fig 2:**
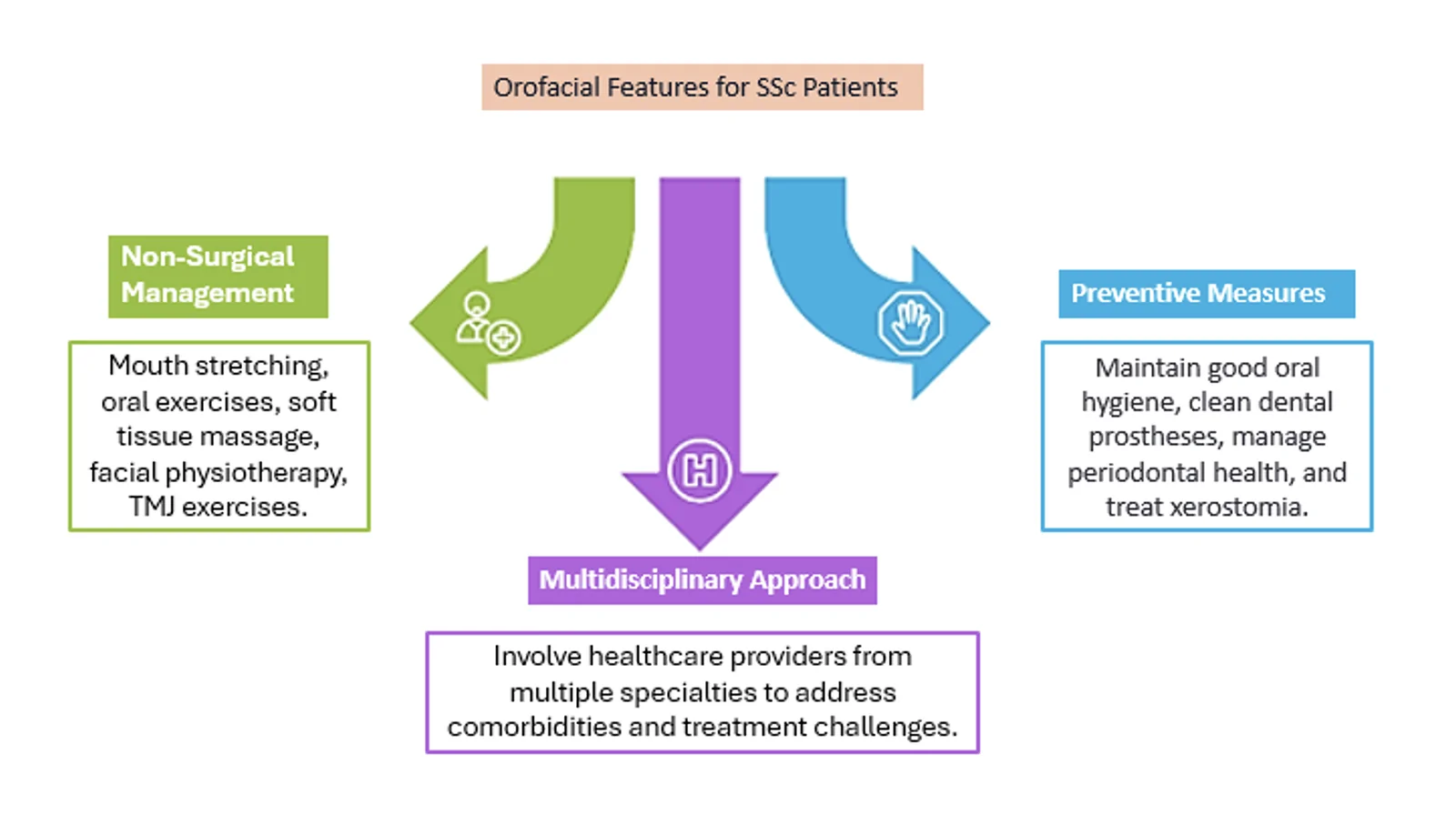
Management of orofacial features in SSc patients.

The literature reports non-surgical management of microstomia using mouth stretching and oral augmentation exercises.^37^ An alternative comprehensive treatment approach includes soft tissue massage techniques, facial physiotherapy, and targeted exercises for the facial muscles, which have demonstrated notable improvements in mouth opening (Fig 2). Connective tissue massage focused on the face and neck aims to enhance blood circulation within the facial soft tissues. Kobat’s technique utilizes neuromuscular activation of the facial muscles alongside active and passive physiotherapy targeting the temporomandibular joint. Research indicates that a multimodal conservative therapy yields better outcomes than any single treatment modality. Given the chronic and progressive nature of the condition, it is advised that patients consistently perform oral exercises to sustain the gains in mouth opening, particularly for those experiencing severe restrictions. Studies have also identified a strong correlation between the severity of the disease and the extent of mouth opening limitation.^7,26,32^

## Diseases Related to Gingival and Periodontal Ligaments (PDL)

Several studies have documented that patients with systemic sclerosis (SSc) exhibit a higher prevalence of gingival and periodontal ligament diseases, with approximately 76% showing increased occurrences of deep periodontal pockets and elevated gingivitis scores.^10^ Periodontal diseases are inflammatory conditions triggered by the buildup of particular bacterial pathogens, leading to the weakening of the host’s immune defences. The relationship between PDL disease and SSc has been found to be linked to decreased salivary production and plaque/calculus deposition. Nevertheless, PDL disease was not directly associated with either the quantity of missing teeth or the extent of disease severity.^7^ Periodontal disease has commonly been assessed by measuring both the periodontal pocket depth and clinical attachment loss. Recent evidence reported a moderate association between SSc and periodontal disease and highlighted that it may cause a degree of alveolar bone loss.^3^ Several studies have found that patients with SSc exhibit a notably high prevalence of PDL disease.^8,42^ Radiological findings among patients with SSc often indicate involvement of the musculoskeletal tissues. Evidence has suggested that this it could be caused by pathological deposition of collagen and/or tightening of adjacent soft tissues.^12,17^ Patients with SSc may show radiological signs including some degree of periodontal ligament widening and different patterns of bone resorption, commonly including the mandibular angle, coronoid process, condylar process, and ascending ramus.^9,11^ Previous reports find that widening of periodontal ligament among patients with SSc is strongly associated with the number of teeth and the degree of severity of SSc. However, no association was found between the number of teeth with PDL widening and either the number of teeth with periodontal disease or the number of missing teeth.^6^ Widening of periodontal ligament in patients with SSc is more common around posterior teeth than anterior teeth.^6^ Interestingly, PDL widening can be associated with different degrees of calcified tissues, such as pulp stones and sclerotic features, leading to obliteration of affected root canals.^23^

As a consequence of fibrotic changes and suspected hypovascularity, the the risk of periodontal and gingival disease may increase, and some degree of recession and/or clinical attachment loss can occur which is mainly associated with posterior teeth.^44^ SSc patients may show a level of hand disability, which can lead to difficulty maintaining good oral hygiene.^14,32^ SSc patients with significant hand impairment may need to use adapted oral hygiene tools to achieve the recommended level of oral health care and reduce the oral health impact of the disease.^43^ Recent studies strongly recommend using appropriately modified interdental cleaning tools and/or flossing, as this can help prevent periodontal disease and interdental lesions.^31,43^ Patients should receive thorough information and guidance on the disease’s risk factors as well as the value of continuing preventative measures, keeping in mind that reduced manual dexterity is a key limitation for the SSc population.^41,43^ Dentists and oral hygienists must emphasize the importance of patients undergoing regular check-ups with their oral health care provider. They should also educate patients about various supplemental oral hygiene tools beyond the standard practices of daily brushing and flossing. Dental prophylaxis with fluoride application has benefits for SSc patients, such as regularly applying 5% sodium fluoride varnish, which can decrease the risk of developing carious lesions.^10,16^

## Dry Mouth and Oral Infection

A significant proportion of patients with SSc, ranging from 32% to 70%, experience xerostomia, making it one of the most common oral symptoms associated with the disease.^4,42^ Xerostomia is commonly linked to fibrosis of the salivary glands, the occurrence of Sjögren’s syndrome, and an elevated accumulation of collagen within the oral mucosal tissues.^8^ Salivary gland hypofunction in SSc patients can give rise to a variety of oral health difficulties, such as increased risk of tooth decay, periodontal disease, halitosis, microbial infections, difficulty in swallowing, and altered taste sensation. Therefore, maintaining good oral hygiene measures should be reinforced while treating SSc patients. Reduced mouth opening, hypofunction of the salivary glands, and negative pharmacological side effects are other oral manifestations of SSc illness that would be expected to increase vulnerability to microbial infection. Low saliva flow rate in SSc patients has been linked to *Candida* infection.^14^ Moreover, there is a considerable chance that individuals using antibiotics, corticosteroids, cyclophosphamide, and/or other immunosuppressants will develop oral candidiasis.^28^ However, education and teaching patients about the importance of maintaining good oral hygiene and appropriate cleaning of dental prostheses, periodontal maintenance, and treatment of xerostomia can help prevent oral infections (Fig 2).^2,10^

Regarding the treatment modalities for oral candidiasis, different forms of antifungal agents can be prescribed such as nystatin, miconazole, and fluconazole, and can be applied in different forms, i.e., as a suspension, tablets, or as a gel directly to the intraoral lesion area and/or inner surfaces of the dental prosthesis. Also, mouthrinses such as chlorhexidine or hexetidine can be beneficial for treating poorly or non-accessible areas.^15^

## Oral Ulcers and Mucosal Atrophy

Atrophy of mucous-membrane linings has been reported among SSc patients, and presents as a pattern of generalized distribution with pathological fibrosis and tissue ischemia that can extend to the gastrointestinal tract.^32^ The clinical presentation of oral mucosa can vary according to the severity of the disease and disease subtype, and may present different degree of dryness and paleness or discoloration.^2^ Complications related to oral mucosal atrophy and oral ulcers can include difficulty speaking and swallowing, as well as oral malodour.^21^ Patients with SSc are often at increased risk of experiencing adverse effects from commonly used treatments. Medications such as cytotoxic and immunosuppressive drugs—including cyclosporine, cyclophosphamide, and methotrexate—as well as antidepressants, antihypertensives, and anticoagulants, may lead to various oral complications such as xerostomia, mucosal ulcers, tissue overgrowth (hyperplasia), and gum bleeding.^13^ Management of related complications can be effective as long as specific symptoms are successfully targeted. Supplements and elimination of local irritants can help decrease disease comorbidities. Subscription of topical corticosteroid agents and antifungal and/or antibacterial therapeutics may be beneficial.^29^

Thus, patients with SSc should be treated by multi-speciality health-care providers, and treatment plans should be aim for a high level of maintaining preventative measures. These should take disease comorbidities and challenges facing both patients and health care providers into consideration. Finally, further research is warranted to explore the nature and impact of SSc and its relation to oral health and periodontal disease.

### CONCLUSION

Systemic sclerosis (SSc) is an immune-driven disorder affecting multiple systems, marked by fibrosis primarily in the orofacial area and hands, which adversely influences oral health and complicates dental care. Based on the observed referral trends and the dental treatments provided, individuals with scleroderma often require the expertise of professionals across multiple dental specialties to address their oral health needs effectively. This review also underscores how the disease can hinder access to dental services. It emphasizes the importance of creating tailored, patient-focused guidelines for oral self-care and ensuring that patients with scleroderma receive timely and suitable dental care to reduce the likelihood of associated oral complications.

### ACKNOWLEDGEMENTS

The preparation of this narrative review was based on a doctoral thesis: “Abdouh I. The impact and nature of oral disease of systemic sclerosis. PhD Thesis, University College London; 2020”. While the thesis was used as background reference material, all text and figures in the present manuscript have been newly written and created for this article. All figures submitted in this manuscript have been created by the author, and the author confirms that each image is original, has not been previously published in whole or in part, and contains no duplicate content. In particular, the section on periodontal manifestations represents original content developed exclusively for this review. Any adaptations drawn from the thesis have been independently rephrased or redrawn and are clearly identified in the figure legends where applicable.
